# A novel cross-validation strategy for artificial neural networks using distributed-lag environmental factors

**DOI:** 10.1371/journal.pone.0244094

**Published:** 2021-01-07

**Authors:** Chao-Yu Guo, Tse-Wei Liu, Yi-Hau Chen

**Affiliations:** 1 Institute of Public Health, National Yang-Ming University, Taipei, Taiwan, R.O.C; 2 Institute of Statistical Science, Academia Sinica, Taipei, Taiwan, R.O.C; South China University of Technology, CHINA

## Abstract

In recent years, machine learning methods have been applied to various prediction scenarios in time-series data. However, some processing procedures such as cross-validation (CV) that rearrange the order of the longitudinal data might ruin the seriality and lead to a potentially biased outcome. Regarding this issue, a recent study investigated how different types of CV methods influence the predictive errors in conventional time-series data. Here, we examine a more complex distributed lag nonlinear model (DLNM), which has been widely used to assess the cumulative impacts of past exposures on the current health outcome. This research extends the DLNM into an artificial neural network (ANN) and investigates how the ANN model reacts to various CV schemes that result in different predictive biases. We also propose a newly designed permutation ratio to evaluate the performance of the CV in the ANN. This ratio mimics the concept of the R-square in conventional statistical regression models. The results show that as the complexity of the ANN increases, the predicted outcome becomes more stable, and the bias shows a decreasing trend. Among the different settings of hyperparameters, the novel strategy, Leave One Block Out Cross-Validation (LOBO-CV), demonstrated much better results, and the lowest mean square error was observed. The hyperparameters of the ANN trained by the LOBO-CV yielded the minimum number of prediction errors. The newly proposed permutation ratio indicates that LOBO-CV can contribute up to 34% of the prediction accuracy.

## Introduction

Numerous studies from different countries have found environmental aspects that are key factors attributable to human mortality [1–4]. Extreme climates occur more frequently than ever due to global warming, encouraging more research on the impact of temperature variations on health outcomes [5, 6]. In addition to temperature, air pollution also plays an important role, such as with CO, O_3_, CO_2,_ and particulate matter (PM) PM_2.5_ and PM_10_ [7, 8]. An air quality report in 2016 indicated that heart disease is a major cause of death in young adults, and the 80% mortality rate is attributable to air pollution [9]. Previously, the Distributed-Lag Non-Linear Model (DLNM) [10] was the ideal strategy to deal with environmental factors that have lag effects such as temperature or air pollution since the cumulative impact could be fitted into the same complex statistical model. DLNM discovered the lag impact of temperature on mortality [11] as well as delayed air pollution [8].

Nowadays, machine learning and artificial neural networks (ANNs) [12] demonstrate superior prediction abilities compared to conventional logistic regression. Applications of the ANNs occurred in many research fields. In particular, an improved fuzzy neural network that predicts traffic speed draws great attentions [13]. Recently, Tan et al. [14] also comprehensively examined both statistical and machine learning methods for incident clearance time prediction. Parameter tuning is crucial in machine learning, and cross-validation (CV) is the primary step for finding the optimal setting of hyperparameters without overfitting, where the tenfold CV is the most popular procedure [15, 16]. The CV error is defined as $\frac{1}{k}\overset{k}{\underset{i=1}{\sum }}(MSE_{\left(k\right)})$, where *MSE*_(*k*)_ is the mean square error (MSE) for the *k*-th-fold dataset, and the smallest CV error suggests the optimal setting of the hyperparameters.

However, CV with time-series data raises a serious issue if each data point is randomly selected and then shuffled without keeping the time sequence. In this case, the later data is used to predict earlier outcomes, which violates the serial pattern assumption. Therefore, h-block cross-validation was proposed [17]. However, this method causes information loss. Later, four strategies for CV were examined by Bergmeir et al. [18] including the fivefold CV, leave-one-out CV, h-block fivefold CV, and out-of-sample evaluation. Among these, the fivefold CV demonstrated the most satisfying results. Following this approach, recent research has focused on error bias estimates using the generalized linear model (GLM) and random forrest (RF) methods [19].

To date, there is no machine learning or artificial neural network considered the most popular artificial intelligence (AI) model that deals with DLNM. Therefore, if lag environmental factors such as temperature or air pollution can be properly incorporated by an ANN, then the predictive model and accuracy are expected to be substantially improved. Therefore, this study aims to develop a novel CV procedure for an ANN that incorporates the complex lag exposure based on the DLNM structure. When CV is conducted in machine learning or artificial neural networks (ANNs), one randomly splits the entire data into 10 unrelated sets. Here, we propose an opposite approach that preserves the correlation owing to the nature of time-series data with predictors and lag effects. We anticipate that the new strategy will outperform the fivefold CV [18].

## Materials and methods

In Taipei City, all-cause daily mortality was obtained from the Cause of Death Database published by the Ministry of Health and Welfare from January 1, 2012, to December 31, 2016. The Institutional Review Board (IRB) of the National Yang-Ming University approved the use of anonymous mortality data and satisfied ethics guidelines. The approved IRB number was YM107045E. Daily mean temperature records were downloaded from the Taipei Weather Station. The freely available data are governed by the Central Weather Bureau (CWB) Observation Data Inquiry System website [20]. Air pollution, including the daily mean ozone concentration and daily mean PM_2.5_ concentration, were downloaded from the Taipei Air Quality Monitoring System, which is maintained by the Environmental Protection Administration Executive Yuan website [21]. Although some air pollutants were missing, we could only omit these observations because the missing rate is low with an ignorable impact on the analyses. Descriptive statistics are listed in Table 1.

**Table 1:** 2012–2016 descriptive statistics for mortality, climate, and air pollution data.

Daily Data		Descriptive Statistics			
	N	Mean	Std Dev	Min	Max
Death	1818	61.39	9.583	32.000	101.000
Temperature	1818	23.57	5.526	5.583	32.996
CO	1816	0.64	0.207	0.080	2.699
O_3_	1816	28.03	9.629	6.317	81.967
PM_10_	1816	39.69	16.802	11.857	142.000
PM_2.5_	1801	20.46	10.412	3.714	87.143
SO_2_	1816	2.89	1.123	0.743	11.571

The DLNM models the current mortality, which is defined as follows:

$$log(\mu_{t})=\alpha +s(x_{t},l,\beta )+\beta_{O_{3}}{O_{3}}_{t}+\beta_{PM2.5}{PM2.5}_{t}+\sum_{i=1}^{p}f({z_{t}}^{i};\;\theta )$$

The independent (predictive) variable (*x_t_*) is the daily mean temperature, and other pollutant variables (${O_{3}}_{t}$ and *PM*2.5_*t*_) are treated as potential confounders. The dependent (outcome) variable (*μ_t_*) was the all-cause daily mortality. The DLNM was fitted through a cross-basis function s(*x_t_,l,β*) that simultaneously describes the effect of the daily mean temperature *x_t_* and its lag structure with maximum lag *l* on the expected daily mortality. Daily mean ozone concentration ${O_{3}}_{t}$ and daily mean concentration *PM*2.5_*t*_ were treated as fixed effects. A natural cubic spline *f*(*z_t_^i^*; *θ*) with eight degrees of freedom for each year was used to adjust for the seasonal effect. In general, the maximum exposure lag *l* was 30. The cross basis consists of a quadratic B-spline for temperature with the knots placed at 10, 75, and 90 percentiles and a natural cubic spline for the lag with 5° of freedom, which indicates three internal knots equally spaced on the log scale.

Instead of the DLNM, we aim to implement an ANN to accommodate such complex structures with a large number of correlated predictors by treating all predictors in the DLNM as input neurons. Previously, mortalities were included as additional input neurons in the ANN. In this way, the ANN could assess whether variations in previous mortality records would affect the mortality outcome in the current day. Therefore, the ANN treats a large set of predictors as different neurons in the input layer, and the training process of the ANN could capture nonlinear associations and provide a satisfying prediction of the mortality outcome. By contrast, the DLNM ignores lag mortality records. Since the compiled dataset is between 2012 and 2016, the year variable is coded as five variables, and the month variable is coded as 12 variables. Weekday, weekend, and holidays are three indicator variables. Up to 30 lags of the temperature variable and mortality were considered by the ANN.

**Fig 1:**
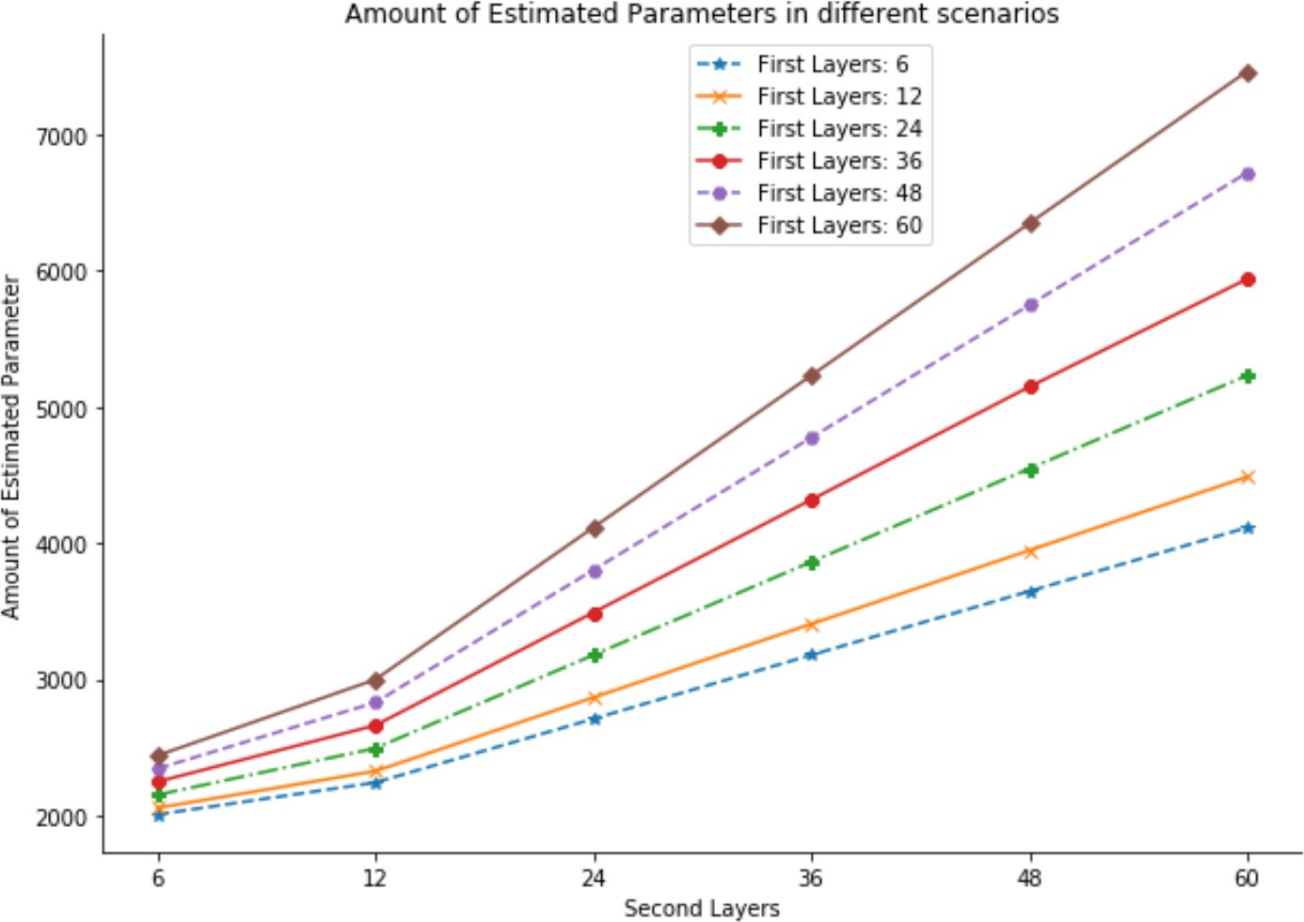
Number of parameters estimated by the ANN.

Regarding the number of hidden layers, the ANN is fitted by two or three layers since one layer may not be suitable for such a complex distributed-lag time series. For an ANN with two hidden layers, the number of neurons was 6, 12, 24, 36, 48, and 60, which resulted in 36 combinations. Regarding an ANN with three hidden layers, owing to the limitation of running time, only three scenarios were considered: (6, 6, 6), (36, 36, 36), and (60, 60, 60). With respect to the number of hidden layers and the number of neurons, the number of parameters to be estimated in the ANN model increases dramatically (Fig 1). The smallest number of parameters is 2,007 for 2 hidden layers, with 6 neurons in each layer. The highest number of parameters is 11,121 when 3 hidden layers with 60 neurons in each layer are trained. Therefore, four or more hidden layers are not practical, and these scenarios were not considered.

**Fig 2:**
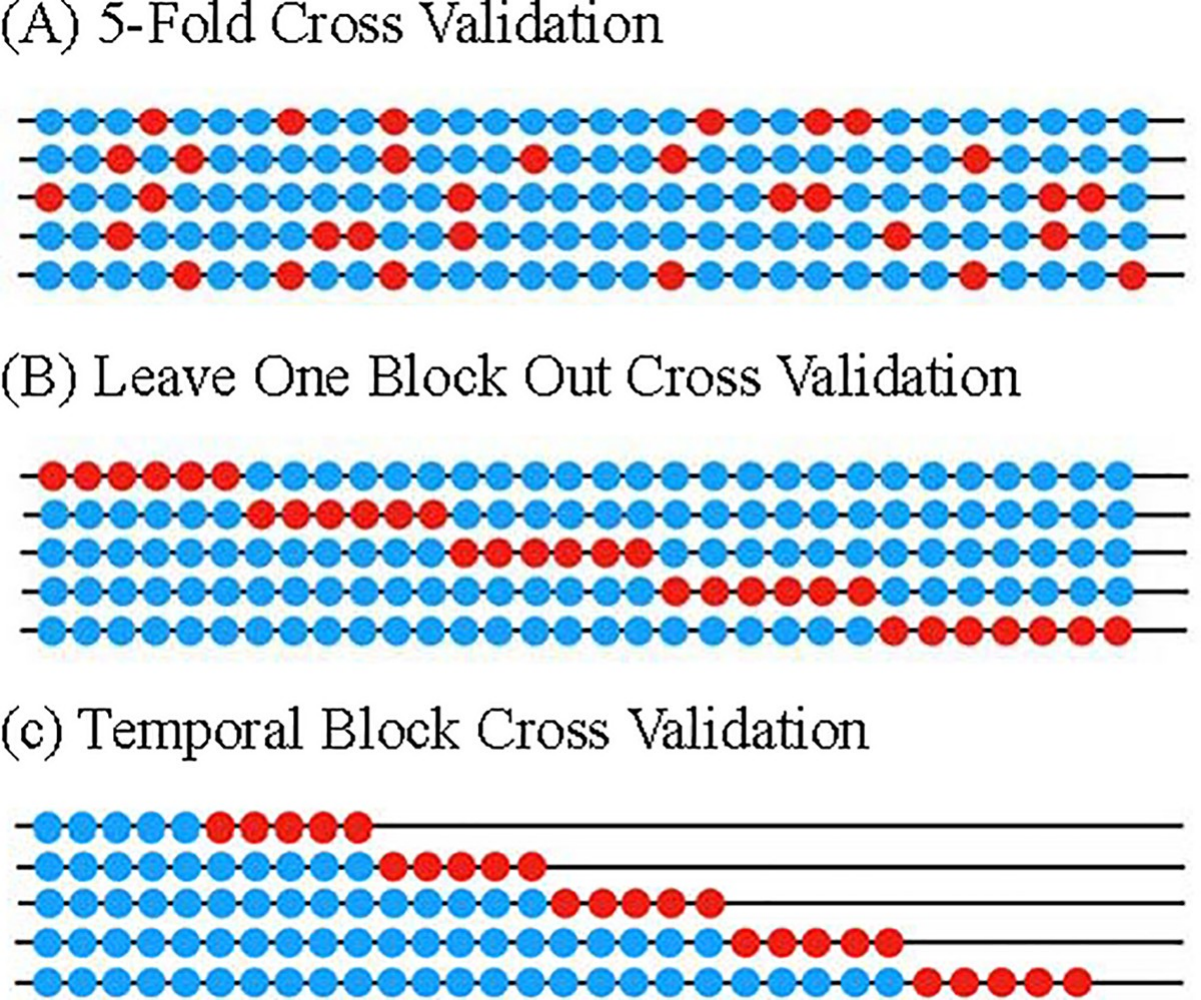
Study designs of the 5-Fold-CV, LOBO-CV, and TB-CV.

According to Bergmeir et al. [18], the fivefold CV was the best performer. Hence, it is the only strategy to be compared with the new methods. The first novel algorithm is Leave-One-Block-Out Cross-Validation (LOBO-CV), and the second is Temporal-Block Cross-Validation (TB-CV). The concept of the three CV schemes is displayed in Fig 2. LOBO-CV utilizes all distributed-lag time series since part of the training set occurred before the testing set. LOBO-CV has a concept similar to that of leave-one-out cross-validation, but each iteration involves a group of time series and results in fewer operations. TB-CV is based on LOBO-CV but avoids unreasonable predictions using the later data. Hence, the sample size of TB-CV is significantly reduced compared to LOBO-CV, especially in the first sequential block.

The preceding 70% of the time-series data is the training set, and the remaining 30% is used as the testing set. In the training set, fivefold CV, LOBO-CV, and TB-CV are implemented. Therefore, the CV error is the average of the five validation errors.

The validity of the three CV methods was further evaluated by a permutation study. We define a new statistic, the permutation ratio (PR), to assess the changes in CV errors from the null hypothesis of no association between mortality and the set of complex correlated predictors to the alternative hypothesis, the observed situation. The CV error with permutation could be compared to that without permutation, which is the original training data.

The PR is defined as

$$PR=min\;(1,\frac{CV\;error(without\;permutation)}{CV\;error(with\;permutation)})$$

The rationale is that if the CV error does not contribute to the prediction accuracy, then the PR would be close to 1. The higher the PR, the lower the impact of the prediction accuracy using ANN. If the PR is close to 0, then the results suggest that the training process of ANN-based on such a CV scheme has the highest impact on prediction accuracy, and all variations are explained by the model. After the best hyperparameter and the optimal CV strategy are determined by the minimum CV error, the ANN can be fitted to the testing dataset and obtain a fair and robust prediction accuracy.

## Results

**Fig 3:**
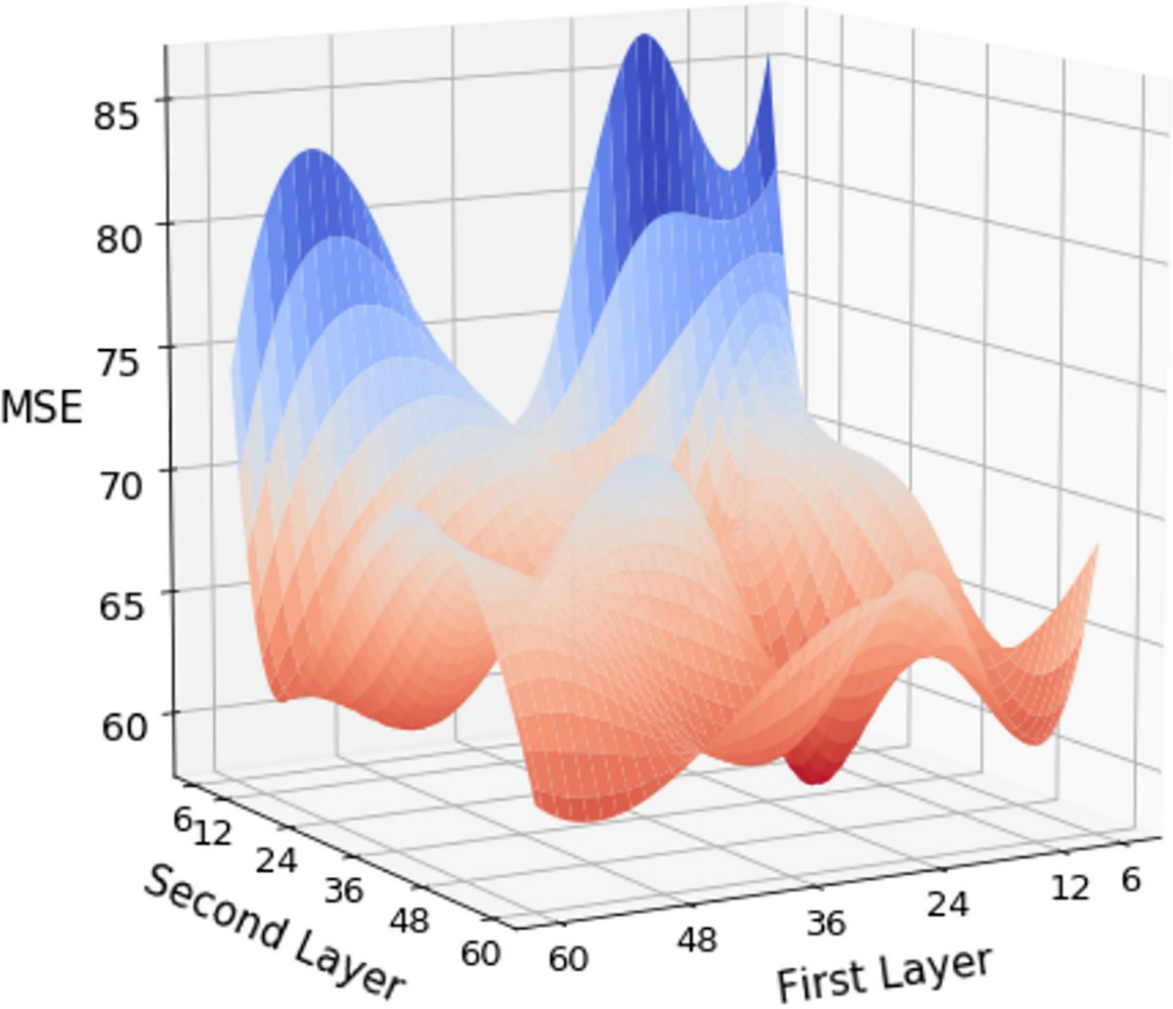
Distribution of MSE for 5-Fold-CV with two hidden layers.

**Fig 4:**
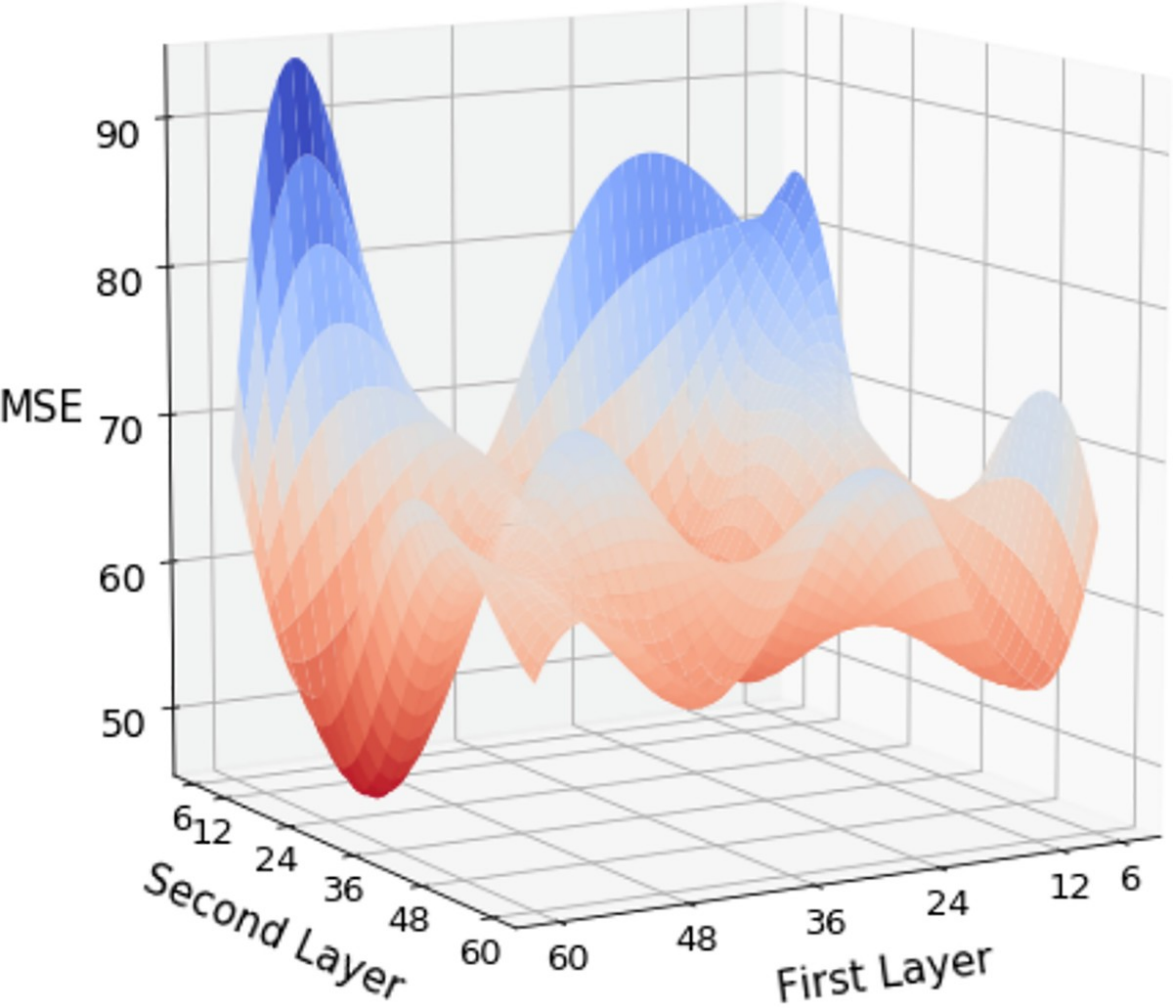
Distribution of MSE for LOBO-CV with two hidden layers.

**Fig 5:**
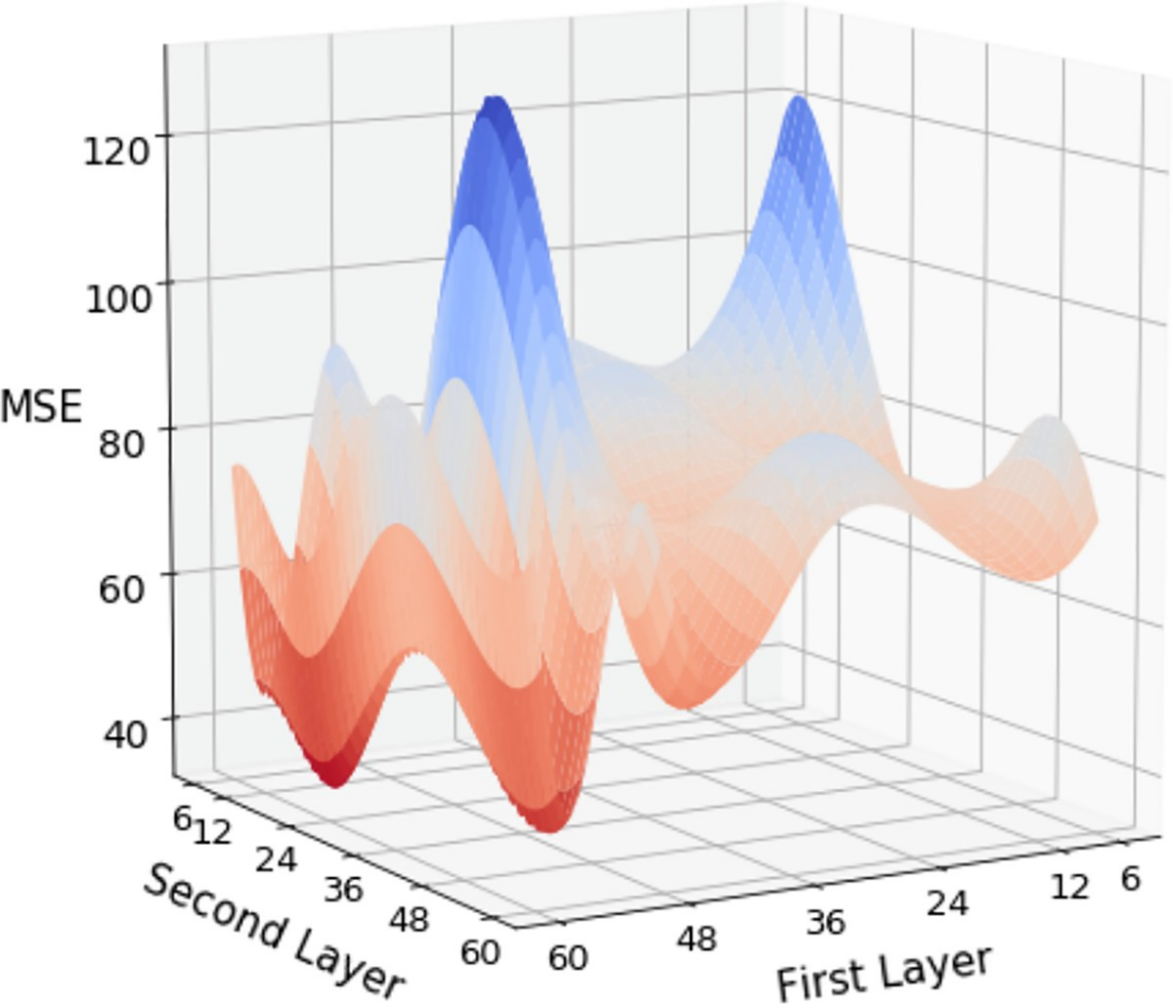
Distribution of MSE for TB-CV with two hidden layers.

In the ANN with two hidden layers, we combined the number of neural nodes in two layers with 6, 12, 24, 36, 48, and 6. Changes in the results were observed under combinations of hyperparameters. In the simulation results of the neural network architecture with two hidden layers, one can observe the preset results under three types of CV with different hyperparameters in Tables 2, 3 and 4. Comparing the results of the three types of CV, we discovered that as the complexity of the model increases, the predicted results improve with a lower MSE. Regarding the three strategies, fivefold CV, LOBO-CV, and TB-CV, the best performing hyperparameter groups are (60, 12), (24, 60), and (36, 6), respectively, where the MSE is 61.192, 54.442, and 65.936, respectively. Among all 36 sets of hyperparameters, the fivefold CV achieves the lowest MSE among the three methods among the eight sets of hyperparameters. The LOBO-CV has 28 sets of hyperparameters, and the lowest MSE is observed. By contrast, TB-CV was not the best performer on any set of hyperparameters. The visualized presentation of all hyperparameters for the three strategies is displayed in Figs 3, 4 and 5, where the trend of decreasing MSE is not linear but has a clear direction.

**Table 2:** CV errors for the 5-Fold-CV.

**Neurons**	**(6,6)**	**(6,12)**	**(6,24)**	**(6,36)**	**(6,48)**	**(6,60)**	**Average**
**CV error**	85.426	81.304	81.338	71.188	81.336	74.154	79.12
**Neurons**	**(12,6)**	**(12,12)**	**(12,24)**	**(12,36)**	**(12,48)**	**(12,60)**	
**CV error**	71.144	72.804	64.592	71.302	71.212	62.352	68.90
**Neurons**	**(24,6)**	**(24,12)**	**(24,24)**	**(24,36)**	**(24,48)**	**(24,60)**	
**CV error**	67.642	70.088	67.496	68.262	61.364	66.178	66.84
**Neurons**	**(36,6)**	**(36,12)**	**(36,24)**	**(36,36)**	**(36,48)**	**(36,60)**	
**CV error**	63.636	67.834	60.954	71.614	67.128	70.878	67.01
**Neurons**	**(48,6)**	**(48,12)**	**(48,24)**	**(48,36)**	**(48,48)**	**(48,60)**	
**CV error**	63.926	63.42	63.758	63.368	67.044	69.514	65.17
**Neurons**	**(60,6)**	**(60,12)**	**(60,24)**	**(60,36)**	**(60,48)**	**(60,60)**	
**CV error**	69.014	61.192	67.588	65.934	61.574	61.466	64.46

Note: the two numbers in parenthesis (a,b) represent the number of nodes in the first (a) and second (b) layer.

**Table 3:** CV errors for the LOBO-CV.

**Neurons**	**(6,6)**	**(6,12)**	**(6,24)**	**(6,36)**	**(6,48)**	**(6,60)**	**Average**
**CV error**	77.44	82.932	82.916	64.442	82.942	67.37	76.34
**Neurons**	**(12,6)**	**(12,12)**	**(12,24)**	**(12,36)**	**(12,48)**	**(12,60)**	
**CV error**	83.714	74.006	59.972	64.262	70.766	58.836	68.59
**Neurons**	**(24,6)**	**(24,12)**	**(24,24)**	**(24,36)**	**(24,48)**	**(24,60)**	
**CV error**	64.904	70.86	56.826	64.104	56.208	54.442	61.22
**Neurons**	**(36,6)**	**(36,12)**	**(36,24)**	**(36,36)**	**(36,48)**	**(36,60)**	
**CV error**	61.646	65.514	53.66	66.154	66.034	67.444	63.41
**Neurons**	**(48,6)**	**(48,12)**	**(48,24)**	**(48,36)**	**(48,48)**	**(48,60)**	
**CV error**	72.152	58.502	62.582	62.962	61.992	66.506	64.12
**Neurons**	**(60,6)**	**(60,12)**	**(60,24)**	**(60,36)**	**(60,48)**	**(60,60)**	
**CV error**	65.538	55.306	69.122	63.274	59.88	59.774	62.15

Note: the two numbers in parenthesis (a,b) represent the number of nodes in the first (a) and second (b) layer.

**Table 4:** CV errors for the TB-CV.

**Neurons**	**(6,6)**	**(6,12)**	**(6,24)**	**(6,36)**	**(6,48)**	**(6,60)**	**Average**
**CV error**	105.074	86.83	86.796	84.622	86.836	75.072	87.54
**Neurons**	**(12,6)**	**(12,12)**	**(12,24)**	**(12,36)**	**(12,48)**	**(12,60)**	
**CV error**	120.94	91.162	81.738	75.194	71.402	71.892	85.39
**Neurons**	**(24,6)**	**(24,12)**	**(24,24)**	**(24,36)**	**(24,48)**	**(24,60)**	
**CV error**	86.708	74.842	81.502	76.46	76.684	71.73	77.99
**Neurons**	**(36,6)**	**(36,12)**	**(36,24)**	**(36,36)**	**(36,48)**	**(36,60)**	
**CV error**	**65.936**	75.728	74.366	72.372	120.144	92.18	83.45
**Neurons**	**(48,6)**	**(48,12)**	**(48,24)**	**(48,36)**	**(48,48)**	**(48,60)**	
**CV error**	82.374	72.404	81.142	74.824	82.146	78.956	78.64
**Neurons**	**(60,6)**	**(60,12)**	**(60,24)**	**(60,36)**	**(60,48)**	**(60,60)**	
**CV error**	73.674	66.702	76.936	69.528	74.32	78.148	73.22

Note: the two numbers in parenthesis (a,b) represent the number of nodes in the first (a) and second (b) layer.

In the case of two hidden layers, the permutation-based CV errors are listed in Tables 5, 6 and 7, where the null hypothesis of no association is simulated. It can be observed that the comparative performance is almost similar, but the MSE increases between 80 and 82 for most sets of hyperparameters. The average MSEs of the fivefold CV, LOBO-CV, and TB-CV are 82.03, 80.016, and 83.279, respectively. The results match our expectation that under the null hypothesis, the three models should yield similar errors, and the errors should be higher than the CV errors of the original data.

**Table 5:** CV errors for the 5-Fold-CV with permutations.

**Neurons**	**(6,6)**	**(6,12)**	**(6,24)**	**(6,36)**	**(6,48)**	**(6,60)**
**CV error**	83.612	81.4	81.404	79.8	79.838	81.4
**Neurons**	**(12,6)**	**(12,12)**	**(12,24)**	**(12,36)**	**(12,48)**	**(12,60)**
**CV error**	**75.81**	94.086	84.55	81.4	76.932	81.378
**Neurons**	**(24,6)**	**(24,12)**	**(24,24)**	**(24,36)**	**(24,48)**	**(24,60)**
**CV error**	84.882	81.396	89.572	84.03	87.98	78.13
**Neurons**	**(36,6)**	**(36,12)**	**(36,24)**	**(36,36)**	**(36,48)**	**(36,60)**
**CV error**	80.996	81.394	81.392	81.4	81.396	81.392
**Neurons**	**(48,6)**	**(48,12)**	**(48,24)**	**(48,36)**	**(48,48)**	**(48,60)**
**CV error**	81.396	80.496	81.394	81.402	83.042	81.396
**Neurons**	**(60,6)**	**(60,12)**	**(60,24)**	**(60,36)**	**(60,48)**	**(60,60)**
**CV error**	81.398	81.398	81.398	81.394	81.402	81.394

Note: the two numbers in parenthesis (a,b) represent the number of nodes in the first (a) and second (b) layer.

**Table 6:** CV errors for the LOBO-CV with permutations.

**Neurons**	**(6,6)**	**(6,12)**	**(6,24)**	**(6,36)**	**(6,48)**	**(6,60)**
**CV error**	79.326	81.838	81.836	80.534	76.284	81.838
**Neurons**	**(12,6)**	**(12,12)**	**(12,24)**	**(12,36)**	**(12,48)**	**(12,60)**
**CV error**	74.936	82.5	82.082	81.83	71.636	81.82
**Neurons**	**(24,6)**	**(24,12)**	**(24,24)**	**(24,36)**	**(24,48)**	**(24,60)**
**CV error**	85.95	81.832	72.872	81.844	69.3	**69.16**
**Neurons**	**(36,6)**	**(36,12)**	**(36,24)**	**(36,36)**	**(36,48)**	**(36,60)**
**CV error**	71.988	81.848	81.85	81.844	82.026	81.828
**Neurons**	**(48,6)**	**(48,12)**	**(48,24)**	**(48,36)**	**(48,48)**	**(48,60)**
**CV error**	81.822	81.578	81.85	81.832	81.826	81.838
**Neurons**	**(60,6)**	**(60,12)**	**(60,24)**	**(60,36)**	**(60,48)**	**(60,60)**
**CV error**	81.854	81.836	81.826	81.826	81.838	81.832

Note: the two numbers in parenthesis (a,b) represent the number of nodes in the first (a) and second (b) layer.

**Table 7:** CV errors for the TB-CV with permutations.

**Neurons**	**(6,6)**	**(6,12)**	**(6,24)**	**(6,36)**	**(6,48)**	**(6,60)**
**CV error**	81.21	82.794	82.792	81.3	**76.752**	82.802
**Neurons**	**(12,6)**	**(12,12)**	**(12,24)**	**(12,36)**	**(12,48)**	**(12,60)**
**CV error**	78.752	78.556	82.784	82.794	84.75	82.792
**Neurons**	**(24,6)**	**(24,12)**	**(24,24)**	**(24,36)**	**(24,48)**	**(24,60)**
**CV error**	90.82	82.794	81.772	82.78	89.164	91.764
**Neurons**	**(36,6)**	**(36,12)**	**(36,24)**	**(36,36)**	**(36,48)**	**(36,60)**
**CV error**	93.57	82.774	82.778	82.798	82.774	82.788
**Neurons**	**(48,6)**	**(48,12)**	**(48,24)**	**(48,36)**	**(48,48)**	**(48,60)**
**CV error**	82.768	82.792	82.778	82.784	82.804	82.782
**Neurons**	**(60,6)**	**(60,12)**	**(60,24)**	**(60,36)**	**(60,48)**	**(60,60)**
**CV error**	82.774	82.772	82.782	82.774	82.802	82.772

Note: the two numbers in parenthesis (a,b) represent the number of nodes in the first (a) and second (b) layer.

After obtaining both the CV error and the permutation-based CV error, the PR is easily calculated, and a lower value represents a better outcome (Table 8). It can be observed that in the context of different sets of hyperparameters, most of the fivefold CV and LOBO-CV demonstrated better results. Among all 36 sets of hyperparameters, the fivefold CV achieved the lowest MSE. In 11 sets of hyperparameters, LOBO-CV is the best performer with 22 sets of the best PRs, and TB-CV has three sets of best performances.

**Table 8:** The Permutation Ratio (PR) in the two hidden layers.

	**(6,6)**	**(6,12)**	**(6,24)**	**(6,36)**	**(6,48)**	**(6,60)**
**5-Fold-CV**	1	0.999	0.999	0.892	1	0.911
**LOBO-CV**	0.976	1	1	0.800	1	0.823
**TB-CV**	1	1	1	1	1	0.907
	**(12,6)**	**(12,12)**	**(12,24)**	**(12,36)**	**(12,48)**	**(12,60)**
**5-Fold-CV**	0.938	0.774	0.764	0.876	0.926	0.766
**LOBO-CV**	1	0.897	0.731	0.785	0.988	0.719
**TB-CV**	1	1	0.987	0.908	0.843	0.868
	**(24,6)**	**(24,12)**	**(24,24)**	**(24,36)**	**(24,48)**	**(24,60)**
**5-Fold-CV**	0.797	0.861	0.754	0.812	0.697	0.847
**LOBO-CV**	0.755	0.866	0.780	0.783	0.811	0.787
**TB-CV**	0.955	0.904	0.997	0.924	0.860	0.782
	**(36,6)**	**(36,12)**	**(36,24)**	**(36,36)**	**(36,48)**	**(36,60)**
**5-Fold-CV**	0.786	0.833	0.749	0.880	0.825	0.871
**LOBO-CV**	0.856	**0.800**	**0.656**	**0.808**	**0.805**	**0.824**
**TB-CV**	**0.705**	0.915	0.898	0.874	1	1
	**(48,6)**	**(48,12)**	**(48,24)**	**(48,36)**	**(48,48)**	**(48,60)**
**5-Fold-CV**	0.785	0.788	0.783	0.778	0.807	0.854
**LOBO-CV**	0.882	0.717	0.765	0.769	0.758	0.813
**TB-CV**	0.995	0.875	0.980	0.904	0.992	0.954
	**(60,6)**	**(60,12)**	**(60,24)**	**(60,36)**	**(60,48)**	**(60,60)**
**5-Fold-CV**	0.848	0.752	0.830	0.810	0.756	0.755
**LOBO-CV**	0.801	0.676	0.845	0.773	0.732	0.730
**TB-CV**	0.890	0.806	0.929	0.840	0.898	0.944

Note: the 2 numbers in parenthesis (a,b) represent the number of nodes in the first (a) and second (b) layer.

The best hyperparameters for the fivefold-CV, LOBO-CV, and TB-CV are (24, 48), (36, 24), and (36, 6), respectively. Therefore, the best PRs are 0.697, 0.656, and 0.705, respectively. The difference between 1 and PR indicates the contribution of the ANN, which means that the trained neural networks based on the three CV strategies contribute 30.3%, 34.4%, and 29.5% of the prediction accuracy, respectively.

In the ANN with three hidden layers, we used (6, 6, 6), (36, 36, 36), and (60, 60, 60) as three sets of hyperparameters to observe the changing patterns (Table 9). The results suggest that the performance of this particular set of hyperparameters (60, 60, 60) is the best. Therefore, the conclusion is consistent that more complex ANN models have better predictive results. The lowest MSE of the fivefold CV, LOBO-CV, and TB-CV were 59.504, 54.762, and 65.626, respectively. It is worth noting that LOBO-CV is the best performer under all circumstances. All MSEs are between 81 and 83, which is similar to the results shown by the two hidden layers but further narrows the range.

**Table 9:** CV errors and the Permutation Ratio (PR) in the three hidden layers.

**CV errors without permutation**	**(12,12,12)**	**(36,36,36)**	**(60,60,60)**
**5-Fold-CV**	67.294	71.294	59.504
**LOBO-CV**	64.918	69.642	54.762
**TB-CV**	76.188	72.49	65.626
**CV errors with permutation**	**(12,12,12)**	**(36,36,36)**	**(60,60,60)**
**5-Fold-CV**	81.4	81.394	81.392
**LOBO-CV**	81.83	81.844	81.834
**TB-CV**	82.79	82.788	82.806
**Permutation Ratio**	**(12,12,12)**	**(36,36,36)**	**(60,60,60)**
**5-Fold-CV**	0.83	0.88	0.73
**LOBO-CV**	0.79	0.85	0.67
**TB-CV**	0.92	0.88	0.79

Note: the three numbers in parenthesis (a,b,c) represent the number of nodes in the first (a), second (b), and third (c) layer.

Finally, for the permutation error, we can observe that the three methods are consistent with this set of hyperparameters (60, 60, 60) as the best result. Therefore, the best PRs for the fivefold CV, LOBO-CV, and TB-CV are 0.79, 0.85, and 0.67, respectively. In summary, the ANN model contributes approximately 21%, 15%, and 33% of the prediction accuracy, respectively. In the simulations for three hidden layers, there is no case in which the PR is greater than 1, as observed in the two hidden layer cases. This means that the three-hidden-layer model is more robust in such a complex data structure.

After the ANN was trained with the optimal hyperparameters, we implemented the three CV strategies in the two-layer hidden layer since the set of parameters is more detailed compared to the three hidden layers. The hyperparameters selected for fivefold CV, LOBO-CV, and TB-CV are (60, 12), (24, 60), and (36, 6), respectively. The MSEs were 116.36, 109.77, and 112.73, respectively. LOBO-CV remains the best performer in the testing dataset, which is consistent with the results of the training dataset.

## Discussion

This research aims to explore the stability and accuracy of the ANN under different CV methods to address lag effects. This study proposed two novel CV strategies and compared their performances to that of Bergmeir et al. [18], which stated that in pure time-series data, the randomly selected K-fold CV is the best. The performance comparisons were completed by computer simulation, and a novel PR value was proposed to evaluate the comparisons, which could be viewed as the R-square in the ANN. These three methods have different levels of trade-offs between the forward-looking bias and the integrity of the data. Therefore, the simulation results are expected to be a reference for the future use of ANNs to predict time-series data with or without lag effects.

In the simulation results, we can see that as the complexity of the hyperparameters increases, a better performance of the ANN model is observed. This trend is consistent either in the two-hidden-layer or three-hidden-layer ANNs.

However, it is worth noting that the best hyperparameters of the model in each layer are often not the points with the highest model complexity in each layer. We believe that this is because we have limited the training times of the neural network to 50,000 times, which may not be the best solution for the model. The lowest point reached the preset number of training sessions. Therefore, the model results may be affected by different starting points, resulting in a jittery decrease in the final predicted MSE rather than strictly decreasing as the model complexity increases.

In addition, we found that as the model complexity increases from two to three hidden layers, the model performs better in terms of stability and prediction accuracy. Taking LOBO-CV as an example, the hyperparameters of two hidden layers are (12, 12), (36, 36), and (60, 60), and the MSEs are 74.006, 66.154, and 59.774, respectively. For three hidden layers at (12, 12, 12), (36, 36, 36), and (60, 60, 60), the MSE becomes 64.918, 69.642, and 54.762, respectively,

Although the performance results for (36, 36, 36) are poor, the overall stability and prediction rate are better than those of the two-hidden-layer model. We believe that this result is due to the complexity of the DLNM. After all, there are 54 variables in the ANN input neurons with 24 independent variables and 30 lag temperature variables. Therefore, when the amount of data is sufficient, with sufficient training times, a more complex model should be more capable of estimating such complex data. Therefore, the three-hidden-layer model exhibits better performance.

Comparing the three CV methods with each other, we found that when the model complexity is low, the fivefold-CV and LOBO-CV methods have their own advantages and disadvantages. However, as the complexity of the model increases, the performance of LOBO-CV is significantly better than fivefold CV; and in the final 39 groups of hyperparameters, 25 groups have the lowest MSE, demonstrating that under the DLNM structure, LOBO-CV is a better strategy for the ANN model. Finally, TB-CV showed poor predictive accuracy in most of the parameter settings. We speculate that this may be due to the loss of important information because the data points after the validation block are eliminated to preserve the temporal structure.

In this study, a total of 39 different hyperparameter scenarios were simulated for CV performance, plus permutation simulations of 39 hyperparameters. Because the ANN needs both forward and backward propagations to enhance accuracy, the time required for model training will be lengthier than that of other common machine learning models such as random forest [22] or Support Vector Machine (SVM) [23].

Taking the parameters (6, 6) of the two-hidden-layer neural network with the lowest model complexity as an example, it takes 34,502 s to complete the three types of CV, which is approximately 10 h. Therefore, when conducting similar studies, researchers may need to consider the time spent by simulations in advance.

## Conclusions

The following items were researched: 1) a new CV scheme that generates the minimum error for an ANN model, 2) a proposed new permutation ratio such that one can interpret the attributable errors reduced by the ANN model, and 3) the first attempt to extend the DLNM into the ANN structure by treating all predictors, including the lag temperatures, as the input neurons. In the extended ANN model, lag mortalities can be included as additional input neurons. In this manner, the ANN can effortlessly utilize lag mortalities compared to the DLNM, which only assesses the variabilities in the current mortality.

The limitations of this research are as follows. Owing to the tremendous amount of computer running time required, the scenarios in the three-layer ANN model were more limited than the scenarios in the two layers. The lag temperatures were used in the ANN model, but air pollution and other factors did not consider lag effects. Although they did not affect the superiority of the new LOBO-CV over previous CV schemes, more complicated input neurons in the ANN would still be informative.

In future studies, a more detailed grid search for the optimal hyperparameters is desirable. In addition, this study has all-cause mortality, but we could not obtain disease-specific deaths. Studies with health outcomes related to temperature or air pollution could contribute more significantly to the clinical applications of this novel LOBO-CV for ANNs.

## Supporting information

S1 File (CSV)
